# DNA methylation subpatterns at distinct regulatory regions in human early embryos

**DOI:** 10.1098/rsob.180131

**Published:** 2018-10-31

**Authors:** Rongsong Luo, Chunling Bai, Lei Yang, Zhong Zheng, Guanghua Su, Guangqi Gao, Zhuying Wei, Yongchun Zuo, Guangpeng Li

**Affiliations:** 1 State Key Laboratory of Reproductive Regulation and Breeding of Grassland Livestock, Inner Mongolia University, Hohhot 010070, People's Republic of China; 2 College of Life Sciences, Inner Mongolia University, Hohhot 010070, People's Republic of China

**Keywords:** early embryos, genomic regions, dynamic DNA methylation, subpatterns

## Abstract

DNA methylation has been investigated for many years, but recent technologies have allowed for single-cell- and single-base-resolution DNA methylation datasets and more accurate assessment of DNA methylation dynamics at the key genomic regions that regulate gene expression in human early embryonic development. In this study, the region from upstream 20 kb to downstream 20 kb of RefSeq gene was selected and divided into 12 distinct regions (up20, up10, up5, up2, 5'UTR, exon, intron, 3'UTR, down2, down5, down10 and down20). The candidate promoter region (TSS ± 2 kb) was further divided into 20 consecutive subregions, which were termed ‘bins’. The DNA methylation dynamics of these regions were systematically analysed along with their effects on gene expression in human early embryos. The dynamic DNA methylation subpatterns at the distinct genomic regions with a focus on promoter regions were mapped. For the 12 distinct genomic regions, up2 and 5'UTR had the lowest DNA methylation levels, and their methylation dynamics were different with other regions. The region 3'UTR had the highest DNA methylation levels, and the correlation analysis with gene expression proved that it was a feature of transcribed genes. For the 20 bins in promoter region, the CpG densities showed a normal distribution pattern, and the trend of the methylated CpG counts was inverse with the DNA methylation levels, especially for the bin 1 (downstream 200 bp of the TSS). Through the correlation analysis between DNA methylation and gene expression, the current study finally revealed that the region bin −4 to 6 (800 bp upstream to 1200 bp downstream of the TSS) was the best candidate for the promoter region in human early embryos, and bin 1 was the putative key regulator of gene activity. This study provided a global and high-resolution view of DNA methylation subpatterns at the distinct genomic regions in human early embryos.

## 1. Introduction

In mammalian reproduction, a sperm fertilizes an egg, and then the zygote undergoes cleavage and develops into a morula; the inner cells of the morula give rise to the inner cell mass (ICM), from which the embryo proper is derived, whereas the outer cells differentiate into the trophectoderm (TE), which gives rise to extraembryonic tissues [1]. This developmental process requires extensive erasure of epigenetic marks, and the dynamics of pre-implantation are driven by the need to reprogram haploid parental epigenomes for the morula to reach a totipotent state in which epigenetic programmes essential for the first-cell lineage commitment and differentiation are established. DNA methylation is the well-studied epigenetic mark and plays multiple critical roles, including transcriptional silencing, genomic imprinting, X chromosome inactivation and repression of transposable elements [2–6]. DNA methylation predominantly occurs on the cytosine in CpG dinucleotides that are stably distributed throughout mammalian genomes [7–9]. Furthermore, DNA methylation is involved in diverse key cellular processes, such as early embryogenesis, stem cell differentiation, regulation of neuronal development and cancer development [9–11]. During early embryogenesis, cell lineage commitments are accompanied by extensive epigenetic remodelling [5,12]. The greatest reductions in DNA demethylation occur in the zygote and the 2-cell stage during pre-implantation. After implantation, remethylation occurs gradually until the original levels are reached [13,14].

In recent years, advanced techniques have rapidly propelled the field of epigenetics forward and have allowed for genome-wide epigenetic analyses of ever-decreasing amounts of biological materials. These techniques have provided insight into global DNA methylation reprogramming at single-base resolution using single cells at multiple developmental stages during early embryogenesis [6,15–19], allowed for refined assessment of DNA methylation dynamics across genomes during specific developmental stages by evaluating specific classes of DNA elements [13,17,20,21], and allowed for methylation comparisons between early mouse and early human embryos [22]. Although the DNA sequences are identical for all of the cells within a body, epigenetic information differs in every cell and is responsible for the maintenance of different cell types within an organism. However, the extents of methylated DNA sequences differ between cell types and developmental stages. Embryos may display similar phenotypes, but with varying methylations on genomic regions associated with cell proliferation and differentiation [23,24]. Comprehensive analyses of genome-wide DNA methylation variations revealed that DNA methylation is not always negatively correlated with gene expression. In fact, DNA methylation of some loci correlated positively with gene expression [25–27], and for these loci many questions remain to be answered. For example, little is known about the quantitative and specific dynamics of genome-wide DNA methylation, and it is still unknown whether DNA methylation of specific genomic subregions changes during early embryogenesis.

Therefore, genome-wide single-cell- and single-base-resolution datasets and more accurate assessments of DNA methylation dynamics could provide an in-depth understanding of the extent of methylation throughout the genome, as well as the DNA methylation of specific genomic regions involved in the regulation of early embryonic development. In this study, the ±20 kb region of RefSeq genes were selected and divided into 12 regions, and then the promoter regions were divided into 20 consecutive subregions, termed ‘bins’. The DNA methylation dynamics of these regions were systematically analysed along with their effects on gene expression. Finally, we mapped the dynamic DNA methylation subpatterns at these genomic subregions. This study provided a high-resolution and global view of DNA methylation subpatterns at the distinct genomic regions in human early embryos.

## 2. Results

### 2.1. The dynamics of global DNA methylation in human early embryos

**Figure 1.**
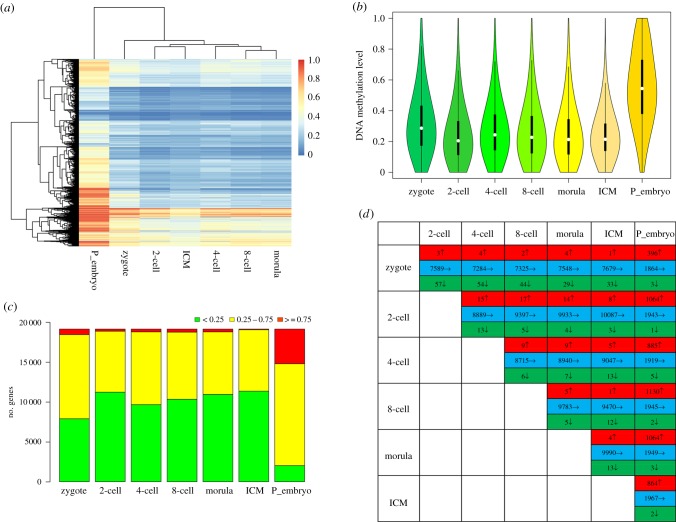
The dynamics of global DNA methylation levels in human early embryos. (*a*) The heat map of DNA methylation of pre- and post-implantation human embryos exhibited two distinct clusters. (*b*) Violin plots of the global DNA methylation dynamics of human embryos. (*c*) Distribution of genes with hyper, moderate or hypo methylation levels at different developmental stages. Red, yellow and green represent genes with hyper, moderate and hypo methylation levels, respectively. (*d*) Differences in the number of hyper- and hypo-methylated genes between pairs of developmental stages. Red, blue and green represent genes whose DNA methylation status went from hypo-methylated to hyper-methylated, maintained hypo-methylation, and went from hyper-methylated to hypo-methylated, respectively. Where the P_embryo is represent the post-implantation embryos.

Analysis of global DNA methylation level indicated two distinct clusters: the pre-implantation stages and the post-implantation stage (figure 1*a*). The DNA methylation level decreased from the zygote to the two-cell stage, remained relatively constant until the ICM of blastocyst, and then dramatically increased at post-implantation stage (figure 1*b*). When DNA methylation level was calculated at the chromosomal level, similar trends were observed (electronic supplementary material, figure S1). To further understand the methylation dynamics of the differentially methylated genes, all the RefSeq genes were classified as hyper-methylated, moderately methylated or hypo-methylated genes. The data showed that most of the genes were moderately methylated or hypo-methylated at the pre-implantation stages. But for the post-implantation embryos, most of the genes were hyper-methylated or moderately methylated. The number of hyper-methylated genes in post-implantation embryos was significantly higher than pre-implantation embryos (figure 1*c*). During the pre-implantation developmental stages, most of the genes maintained their hypo-methylation statuses, only a few genes changed from a hypo-methylated state to a hyper-methylated state or from a hyper-methylated state to a hypo-methylated state. When the methylation states of embryos at any of the pre-implantation stages were compared to the post-implantation stage, 396–1130 genes changed from the hypo-methylated state to the hyper-methylated state, whereas only a few genes changed from the hyper-methylated state to the hypo-methylated state (figure 1*d*).

### 2.2. DNA methylation dynamics at the 12 distinct genomic regions

**Figure 2.**
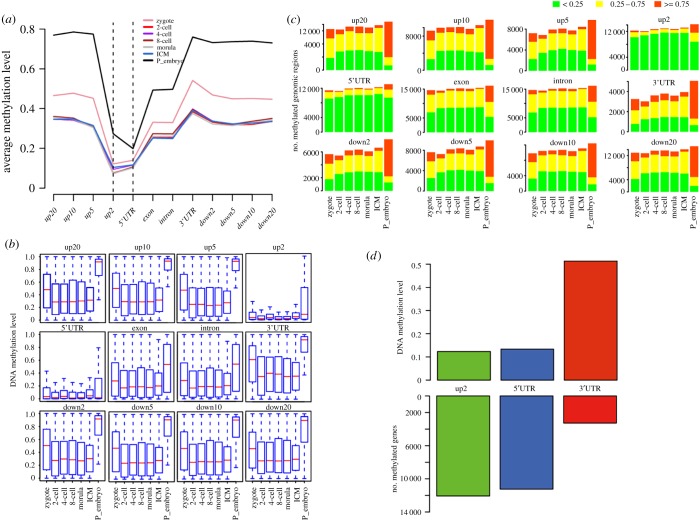
The characteristics of DNA methylation level at the 12 genomic regions in human early embryos. (*a*) Average DNA methylation levels of the 12 genomic regions for each developmental stage. (*b*) Box plots showing the DNA methylation dynamics for each genomic region. (*c*) The number of genes with hyper, moderate or hypo methylation levels at the 12 genomic regions. (*d*) The numbers of methylated genes and DNA methylation levels at the genomic regions up2, 5'UTR and 3'UTR.

To gain insight into the dynamics of DNA methylation at the 12 genomic regions, we analysed the distribution of DNA methylation across CpG clusters within a certain region. From up20 to down20, all the developmental stages shared the same DNA methylation trend, the changes of DNA methylation levels were first decreased then increased gradually (figure 2*a*). For the intergenic regions up20 to up2 and down2 to down20, the up2 region had the lowest DNA methylation level, whereas the other intergenic regions had relatively equivalent DNA methylation levels. For the gene body regions, the DNA methylation level was the lowest at the 5'UTR and the highest at the 3'UTR. However, the DNA methylation levels of the exon and intron were nearly identical (figure 2*a*,*b*). In addition, the DNA methylation dynamics of these regions (except up2 and 5'UTR) were similar to the global DNA methylation dynamics (figures 1*b* and 2*b*).

Since all the embryos at different developmental stages had similar DNA methylation dynamics from up20 to down20, we then classified the genes into hyper-, moderate- and hypo-methylated states according to the DNA methylation level of each region. The result showed that the up2 and 5'UTR regions were hypo-methylated in all the developmental stages. All the other regions were moderate- or hypo-methylated in pre-implantation embryos, and the numbers of moderate- or hypo-methylated genes did not vary significantly. However, in post-implantation embryos, these regions were almost hyper-methylated (figure 2*c*). Notably, the up2 and 5'UTR had the high CpG counts with low DNA methylation levels, but it was totally the opposite for the 3'UTR (figure 2*d*).

### 2.3. DNA methylation dynamics at the 20 consecutive bins within the promoter region

**Figure 3.**
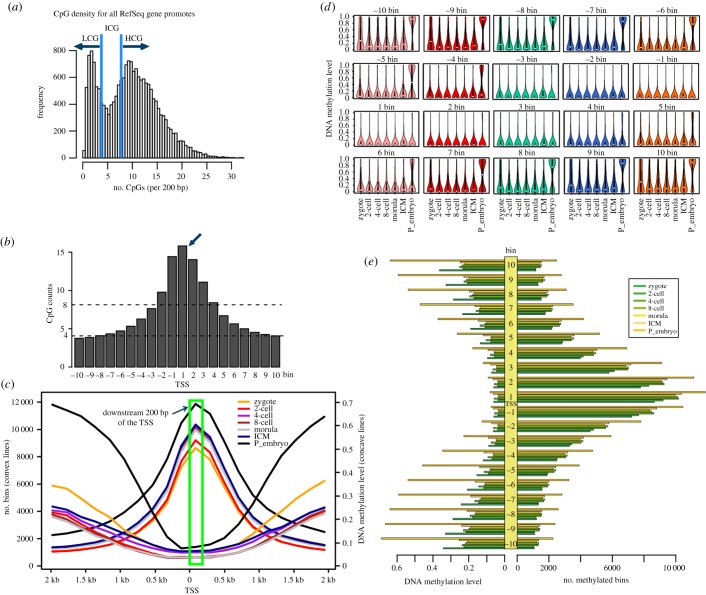
The CpG densities and DNA methylation level of the 20 consecutive bins within promoter region. (*a*) The classification of the CpG densities for all the RefSeq gene promoters. HCG, more than 8 CpG per 200 bp. LCG, less than 4 CpG per 200 bp. ICG, 4–8 CpG per 200 bp. (*b*) The distribution of CpG counts at the 20 bins. (*c*) A line chart showing the status of DNA methylation during human early embryonic development. (*d*) Dynamics of DNA methylation at the 20 bins. (*e*) Comparison of the numbers of methylated bins and the corresponding DNA methylation levels.

DNA methylation of the promoter region regulates gene expression. In most cases, DNA methylation leads to gene silencing. To obtain detailed information regarding DNA methylation in promoter region, the TSS ± 2 kb was defined as the promoter region and was subdivided into 20 consecutive bins, which were classified into three subtypes—high, intermediate and low CpG (HCG, ICG, and LCG, respectively)—based on their CpG density according to the reference sequence (figure 3*a*; electronic supplementary material, figure S2). The data showed that bin −10 and bin −9 were LCG and the other bins were HCG (bin −2, −1, 1, 2, 3, 4) or ICG (the rest of bins). Notably, bin1 which is 200 bp downstream of the TSS had the highest CpG density (figure 3*b*). Then we pooled and counted the number of methylated bins (had methylated CpGs in a certain bin), and the corresponding DNA methylation level were calculated. For the three subtypes of bins, the HCG bins had the lowest DNA methylation levels, while the ICG bins had moderate DNA methylation levels and LCG bins had the highest DNA methylation levels (electronic supplementary material, figure S4).

The CpG counts of the 20 consecutive bins displayed a regular pattern that increase firstly and then decrease. We then asked whether the CpG counts were associated with the methylation level. The results indicated that the trend of the CpG counts was consistent with the number of methylated bins but was inverse with its DNA methylation levels (figure 3*b*,*c*). For the 20 bins, bin −3 to 5 were hypo-methylated in all developmental stages (figure 3*d*), while the other bins had the similar DNA methylation dynamic with the dynamics of genome-wide and the 10 distinct genomic regions (figure 1*b*, 2*b*, 3*d*). All the 20 consecutive bins had similar DNA methylation features for each developmental stage (figure 3*e*). These data indicated that in human early embryos, those bins in promoter region with higher CpG densities had lower DNA methylation levels, whereas the bins with lower CpG densities had higher DNA methylation levels. Moreover, the DNA methylation dynamics in different tissues had the same pattern in these bins (electronic supplementary material, figure S3).

### 2.4. Relationships between DNA methylation at distinct genomic regions and gene expression

**Figure 4.**
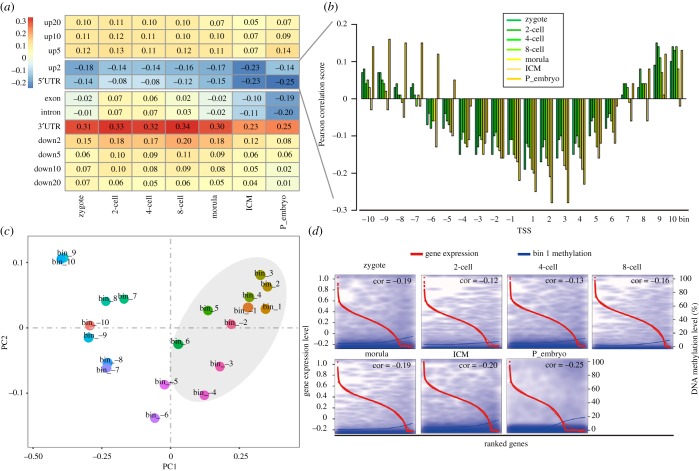
The relationships between DNA methylation level at distinct genomic regions and gene expression. (*a*) The relationships between the DNA methylation level of the 12 genomic regions and gene expression. (*b*) The relationships between the DNA methylation level and gene expression for the 20 consecutive bins. (*c*) PCA showed that the bins from −4 to 6 cluster to gather as depicted by the grey area. (*d*) A scatter plot shows the DNA methylation level and the relative expression level for bin1 of the RefSeq genes. The red and blue curves represent gene expression levels and DNA methylation levels, respectively. Pearson correlation coefficients are marked at the top right corner of each panel. The horizontal axis from left to right below each box represents the RefSeq gene expression level from high to low, respectively.

To gain high-resolution information about DNA methylation and regulation of gene expression, the correlations between DNA methylation at the distinct genomic regions and gene expression were analysed along with previously published single-cell transcriptome data [28]. For the gene body regions, the DNA methylation levels and gene expression were negatively correlated in 5′UTR, but positively correlated in 3'UTR. For exon and intron, the DNA methylation levels were positively correlated with gene expression in two-cell, four-cell and eight-cell stages, and negatively correlated in the zygote, morula, ICM and post-implantation stages. It was noteworthy that the correlations were weak from zygote to morula stages. For the intergenic regions, the DNA methylation levels and gene expression were negatively correlated in up2 but positively correlated in the other regions (figure 4*a*; electronic supplementary material, table S1).

We further evaluated the correlations between DNA methylation and gene expression at those bins in promoter region. The results showed that DNA methylation of bins −4 to 6 were negatively correlated with gene expression (figure 4*b*). The principal component analysis (PCA) also demonstrated that these bins clustered together (figure 4*c*). But this situation was not so suitable for somatic tissues (electronic supplementary material, table S1).

### 2.5. Gene ontology analysis of differentially methylated genes

Considering bin 1 had the highest CpG density and the lower DNA methylation level, there was a strong negative correlation between DNA methylation and gene expression in all the developmental stages (figure 4*d*). Thus, bin 1 was selected for further analysis and the GO analysis was conducted with the differentially methylated genes within bin 1. A total of 363 genes demonstrated decreased DNA methylation and increased gene expression from ICM to post-implantation embryos (electronic supplementary material, table S2). These genes were enriched for terms associated with cell proliferation and differentiation which could better reflect the transition from pre-implantation to post-implantation, such as cell development, organogenesis, proton transporting and response to hypoxia (electronic supplementary material, table S3).

Since all the developmental stages shared the same DNA methylation trend from up20 to down20, we then analysed CD81 gene, which codes for a protein that mediates signal transduction and plays important role in the regulation of cell development, activation, growth and motility [29]. We highlighted the DNA methylation dynamics at the ±20 kb region of CD81 and performed a more detailed analysis of the region 200 bp downstream of the TSS during all the developmental stages. Similar dynamics of DNA methylation were observed compared with above-mentioned results. Finally, a comprehensive landscape was mapped to demonstrate the DNA methylation levels and the correlations between DNA methylation and gene expression in all the regions and bins in human early embryos (electronic supplementary material, figure S5).

## 3. Discussion

DNA methylation has been investigated for many years and is generally considered as a silencing epigenetic mark that regulates gene expression and maintains genome stability [2,30–33]. With the development of high-throughput sequencing technology, genome-wide DNA methylation patterns can be mapped, which facilitates research on the biological functions of DNA methylation in embryonic development, cell differentiation and cancer development.

In present study, we performed an integrated analysis between DNA methylation at distinct genomic regions and gene expression in human early embryos, and mapped DNA methylation patterns at these distinct genomic regions. DNA methylation levels have been shown to correlate with CpG density within distinct genomic regions [34]. The 5'UTR and up2 regions have higher CpG densities and relatively low DNA methylation levels, and are negatively correlated with gene expression. It has been suggested that DNA methylation of the exon and the intron may affect regulation of alternative transcription, exon usage and splicing [35,36]. This analysis indicated that the DNA methylation of the exon and the intron were both relatively stable and correlated weakly positively or negatively with gene expression, thus they may play the same roles in human early embryos. The 3'UTR that flanks the TES is a region where microRNAs bind to regulate gene activity [37–40]. In our study, the 3'UTR had the highest level of DNA methylation and showed a strong positive correlation with gene expression, indicating that the hyper-methylation of the 3'UTR is a feature of transcribed genes. Therefore, the DNA methylation of 3'UTR could be used as a reporter to determine whether a gene is actively transcribed or not. Previous studies suggested that DNA methylation in intergenic regions regulates microRNA expression [41] and that high DNA methylation levels in intergenic regions stabilizes the genome [36]. The DNA methylation levels of the intergenic regions except up2 were relatively high in present study, which may contribute to genomic stability and conservation. Our results regarding the distribution of DNA methylation are consistent with the idea that DNA methylation contributes an additional level of stability to the epigenetic status, and also indicate that the role of DNA methylation has region-specific effects.

As a crucial regulator of gene expression, DNA methylation at the promoter region was thought to suppress gene expression, whereas DNA methylation in the vicinity of the TSS was thought to block transcription initiation [27,28,42,43]. However, the gene promoter region is a fuzzy concept with varied definitions [13,22,44]. In present study, the 20 consecutive bins within the promoter region had high CpG densities, but exhibited low DNA methylation levels, especially for bins −4 to 6 (800 bp upstream to 1200 bp downstream of the TSS). The DNA methylation levels of these bins were negatively correlated with gene expression during all the developmental stages, indicating that this region was essential for regulating gene expression. This region may be used as the best candidate for the promoter region in human early embryonic development. Furthermore, this situation was not completely suited to different tissues in our study; more data should be added for further comparative analysis. GO enrichment analysis were conducted with the differentially methylated genes identified by the bin 1 between the ICM of blastocyst and post-implantation showed that the enriched genes played key roles in the pre-implantation to post-implantation transition. A previous study indicated that methylation of the region downstream of the promoter may regulate usage of alternative promoters [45]. Since the bins located downstream of the TSS are part of the first exon, the first exon may play an important role in regulating gene activity in human early embryos.

Recently, several genomic chromatin researches for mammalian early embryonic development have been published [46–48]. Gao *et al*. [49] mapped the chromatin accessibility landscape in human early embryos for the first time, from the perspective of development and evolution to understand human chromatin in human early embryonic development. They analysed the correlations between DHSs and DNA methylation, and their results showed that high CpG promoter regions tend to establish DHSs, whereas just opposite in the low CpG promoter regions [49]. The integrated analysis between DNA methylation and chromatin accessibility based on the same different genomic regions might increase our understanding of epigenetics.

The epigenetic remodelling that occurs during early embryonic development is complex and dynamic. With the development of new technologies and the use of big data, we can perform integrated multi-omics analysis in the future, including DNA methylation, histone modifications, chromatin accessibility and gene expression, to explore lineage- and species-specific epigenetic remodelling from multiple dimensions to improve our understanding of early developmental events. This study demonstrated a method to detect specific methylation across distinct genomic regions and provided a better understanding of DNA methylation dynamics in human early embryonic development and reprogramming.

## 4. Methods

### 4.1. Data collection and processing

Human genome assembly (hg19) and gene annotations were downloaded from the UCSC genome browser (http://genome.ucsc.edu/). For genes that have alternative transcripts with the same TSS, only one transcript was chosen, and genes that begin with the mature messenger RNA (MM) were analysed further. Genome-wide DNA methylation datasets at single-cell and single-base resolution of human early embryos at pre-implantation (zygote, 2-cell, 4-cell, 8-cell, morula and ICM of blastocyst), post-implantation stage and different tissues (somatic cell at 7 weeks, 10 weeks and 19 weeks, liver at 6 weeks, PGC at 11 weeks) were downloaded from the NCBI Gene Expression Omnibus (GEO) under accession number GSE49828 and GSE63818 [13,21]. The datasets for each developmental stage were composed of one to three biologically replicated samples.

### 4.2. Annotation of genomic regions

**Figure 5.**
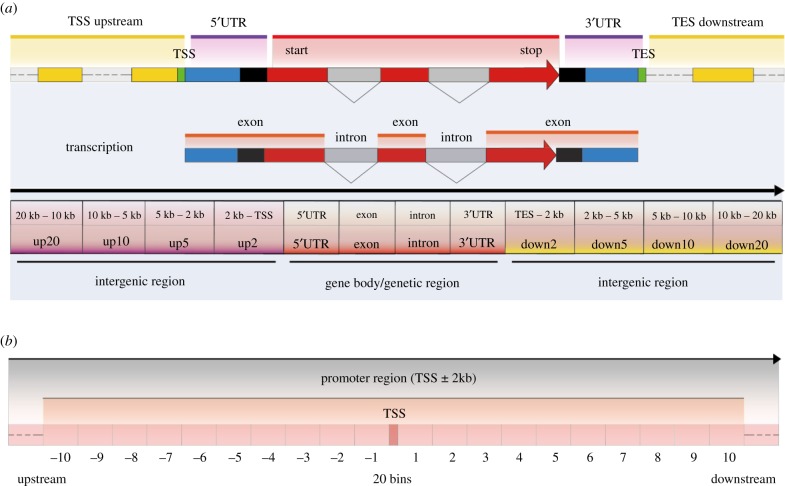
Overview of the distinct genomic regions and the bins within promoter region. (*a*) The region from 20 kb upstream to 20 kb downstream of RefSeq gene was selected and divided into 12 distinct regions, including the gene body region, which we termed the 5'UTR to 3'UTR regions, and the intergenic region, which we termed the up20 to up2 and the down2 to down20 regions. (*b*) The TSS ± 2 kb was defined as candidate promoter region and was further divided into 20 consecutive subregions, termed bins.

To evaluate DNA methylation dynamics at distinct genomic regions in human early embryos, the region ±20 kb of RefSeq genes was selected and divided into gene body and intergenic regions based on gene structure and annotations. The gene body—the genomic region from TSS (transcript start site) of a gene to its TES (transcript end site)—was composed of the 5'UTR (untranslated region), exon, intron and the 3′ UTR. In this study, the ‘exon’ includes all the exons within a gene and so does the ‘intron’. The intergenic region was defined as the complement of the gene body and comprises the upstream 20 kb and the downstream 20 kb of the gene. The intergenic region was then divided into eight regions, including the region 20 kb upstream of the TSS (up20) and the region 0 kb downstream of the TES (down2) and so on. Finally, the DNA methylation dynamics of the 12 distinct genomic regions of the RefSeq genes were analysed individually (figure 5*a*).

To explore the detailed DNA methylation dynamics of the promoter region, the TSS ± 2 kb region was defined as the promoter region and further binned it into 20 consecutive bins (200 bp for each) based on gene structure and annotations (figure 5*b*). The number of CpG dinucleotides in each bin was counted. According to the CpG counts, the bins were classified as HCG, ICG and LCG based on thresholds described previously [10].

### 4.3. Integrated analysis of genome-wide DNA methylation and gene expression

The DNA methylation of the gene body and intergenic regions were summed to determine the global DNA methylation levels of the RefSeq genes. Upon calculation of the DNA methylation levels of these genomic regions, only the CpG sites with read coverage greater than five were analysed further. The average of the DNA methylation level of all the CpGs captured in a region was interpreted as the average DNA methylation level of the region. Analysis of DNA methylation of single CpG was also limited to the CpGs with at least fivefold read coverage. We defined the DNA methylation level of each sample as the arithmetic average of the DNA methylation levels of all the RefSeq genes, whereas the DNA methylation level of each reported developmental stage was defined as the arithmetic average of all the biological replicates.

An integrated analysis of DNA methylation and gene expression was conducted to assess the relationship between DNA methylation and gene expression. The log2 of gene expression levels (reads per kilobase of transcript per million mapped reads, RPKM) of RefSeq genes was calculated, and genes with an RPKM equal to 0.0 were reset to the minimum value of non-zero. The Pearson correlation coefficient (r) between gene expression and DNA methylation was calculated for each genomic region and bin. The genome-wide single-cell gene expression data of human early embryos and different tissues were used for an associated analysis with DNA methylation. The data calculations and the integrated analysis were performed using customized Python scripts.

### 4.4. Functional annotation of differentially methylated genes

GO analysis was conducted for the following genes: (i) the DNA methylation level of bin 1 decreased more than twofold from ICM to post-implantation (*t*-test, *p* < 0.5) and for genes in which the absolute DNA methylation value was greater than the mean DNA methylation value; (ii) the gene expression level increased from ICM to post-implantation. The GO analysis was performed with DAVID online (http://david.abcc.ncifcrf.gov/) [50]. A representational item was defined as a modified Fisher's exact *p*-value with an adjustment for multiple tests using the Benjamini method. Data analysis and visualization were performed using customized R scripts.

## Supplementary Material

figure S1

## Supplementary Material

figure s2

## Supplementary Material

figure S3

## Supplementary Material

figure S4

## Supplementary Material

figure S5

## Supplementary Material

table S1.doc

## Supplementary Material

table S2.doc

## Supplementary Material

table S3.doc

## Data accessibility

All the DNA methylation data and gene expression data were deposited at the NCBI Gene Expression Omnibus (GEO) under accession number GSE49828, GSE63818 and GSE36552.
