# Correction to ‘DNA methylation subpatterns at distinct regulatory regions in human early embryos’

**DOI:** 10.1098/rsob.180215

**Published:** 2018-12-05

**Authors:** Rongsong Luo, Chunling Bai, Lei Yang, Zhong Zheng, Guanghua Su, Guangqi Gao, Zhuying Wei, Yongchun Zuo, Guangpeng Li

*Open Biol.* **8**, 180131. (Published online 31 October 2018). (doi:10.1098/rsob.180131)

The *p*-value of significance test has been given as ‘*p* < 0.5’ in the Material and methods section (4.4), which is incorrect.

The correct writing for the *p*-value is ‘*p* < 0.05’. It should be noted that we correctly wrote the *p*-value in the electronic supplementary materials and in responding to the reviewers' comments, i.e. ‘*p* < 0.05’.

All the authors gave their consent to this correction.

