# Supplementary figures and images for: DNA methylation subpatterns at distinct regulatory regions in human early embryos
Source: Open Biol. 2018 Oct 31;8(10):180131. doi: 10.1098/rsob.180131 (PMC6223221; doi:10.1098/rsob.180131)

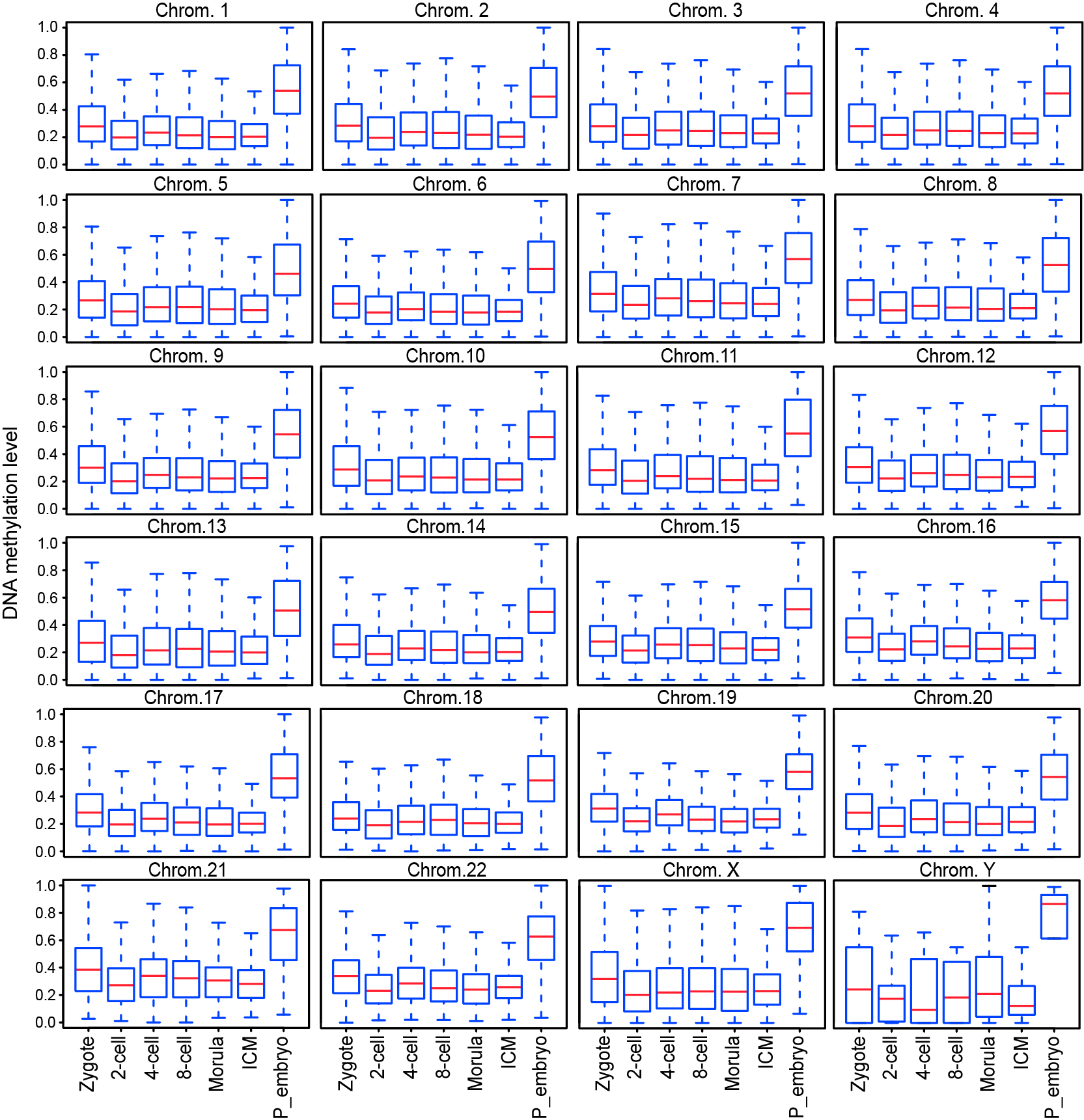

Supplement: figure S1 [file rsob180131supp1.tif]

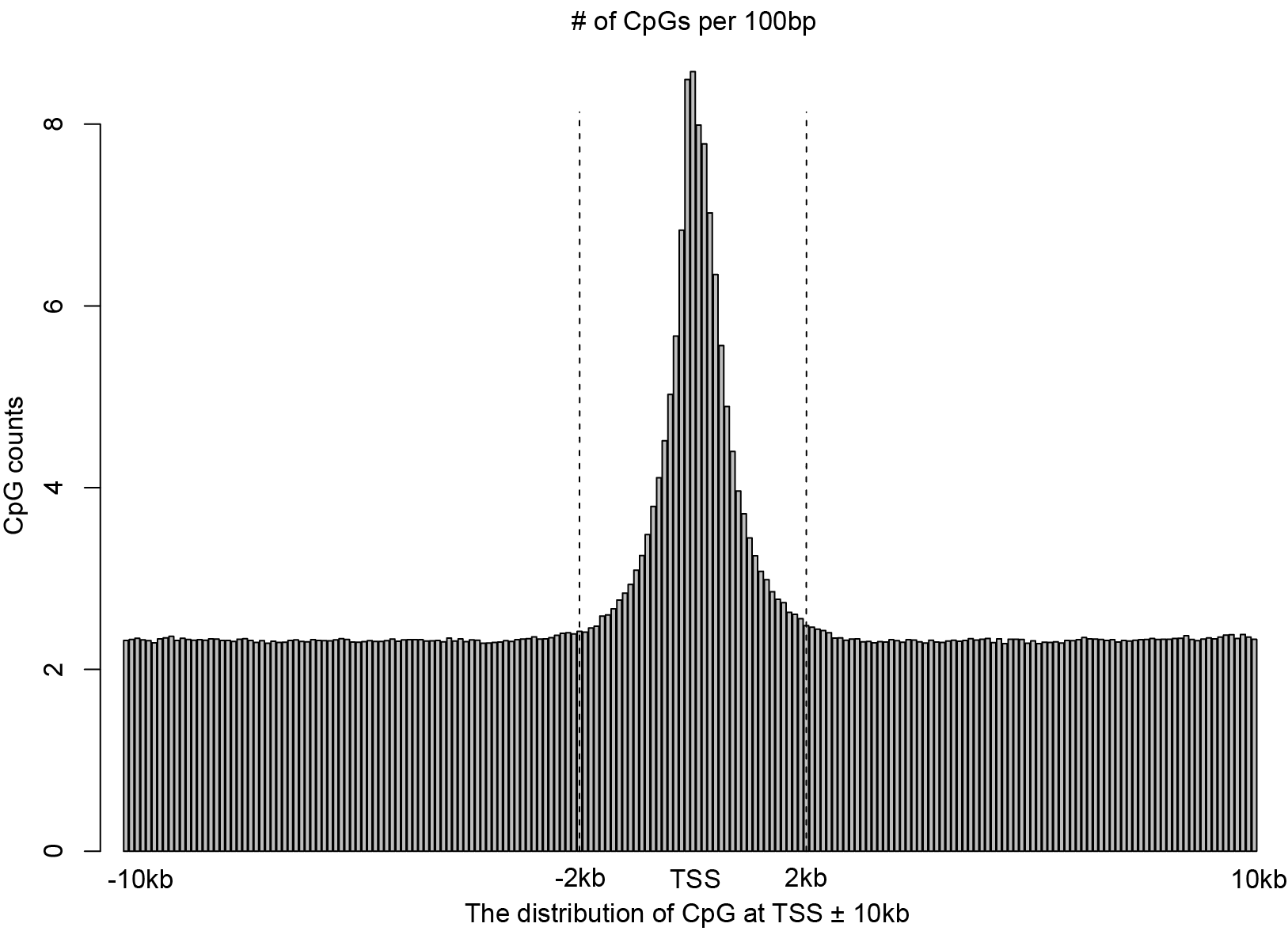

Supplement: figure s2 [file rsob180131supp2.tif]

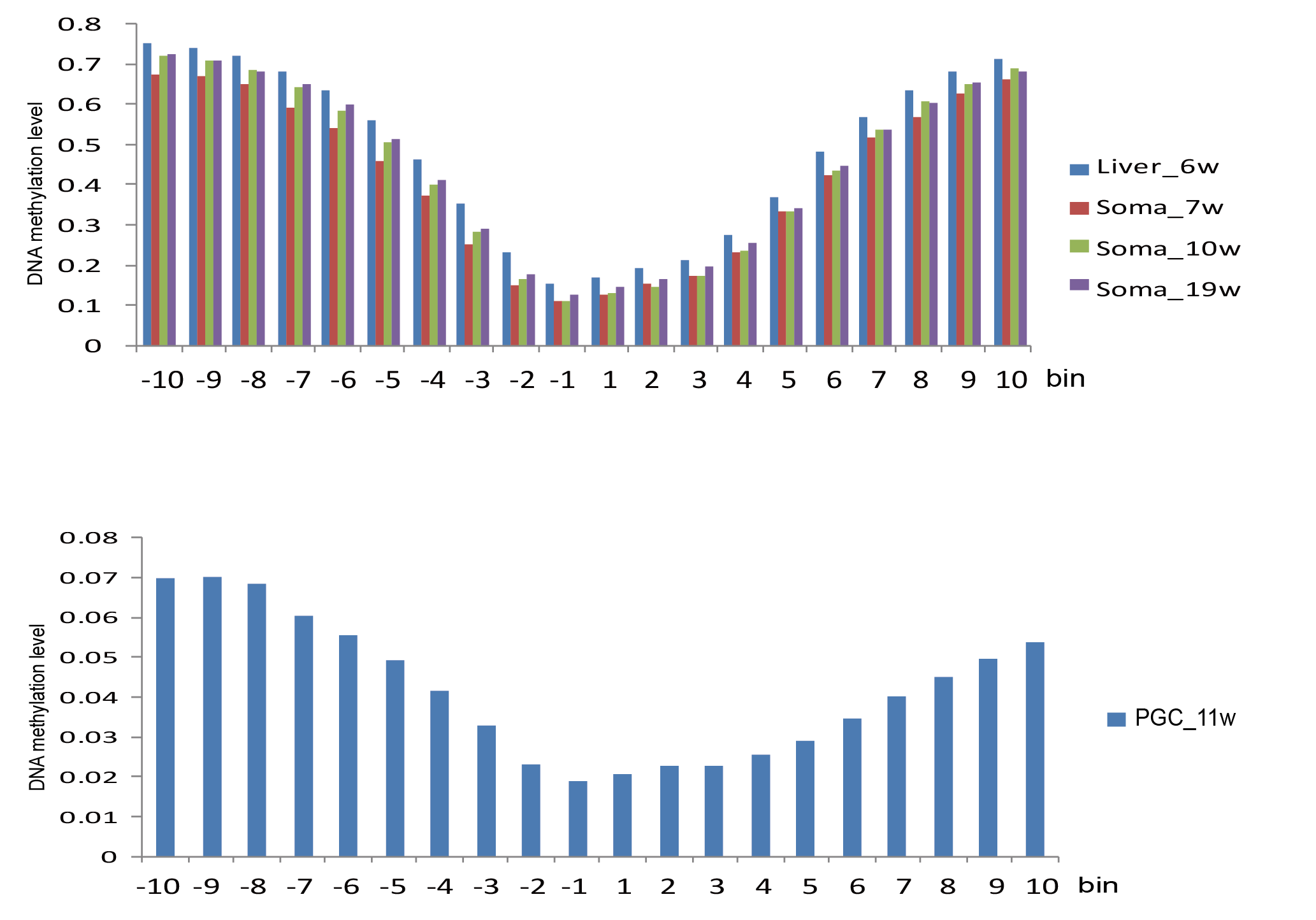

Supplement: figure S3 [file rsob180131supp3.tif]

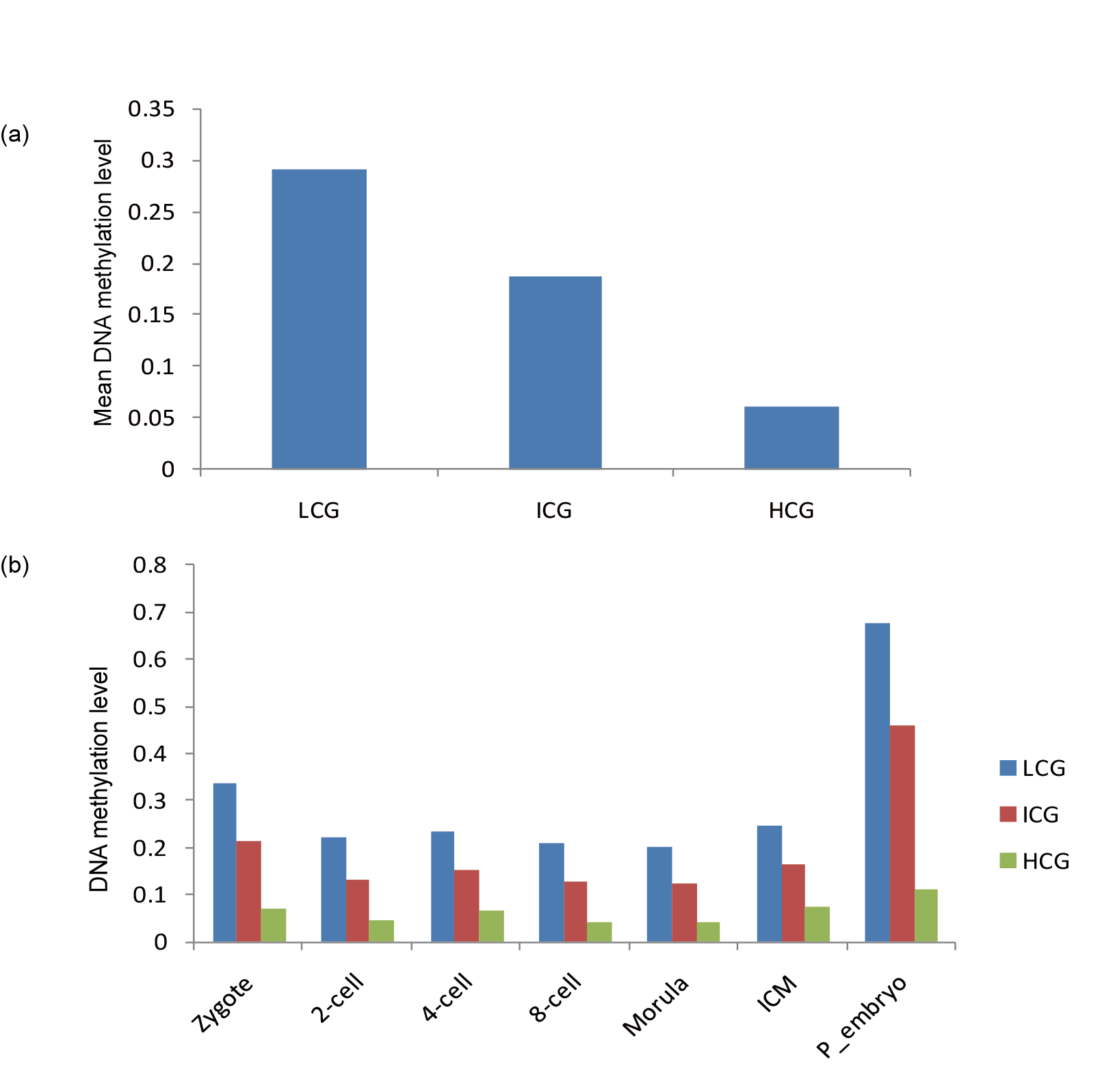

Supplement: figure S4 [file rsob180131supp4.tif]

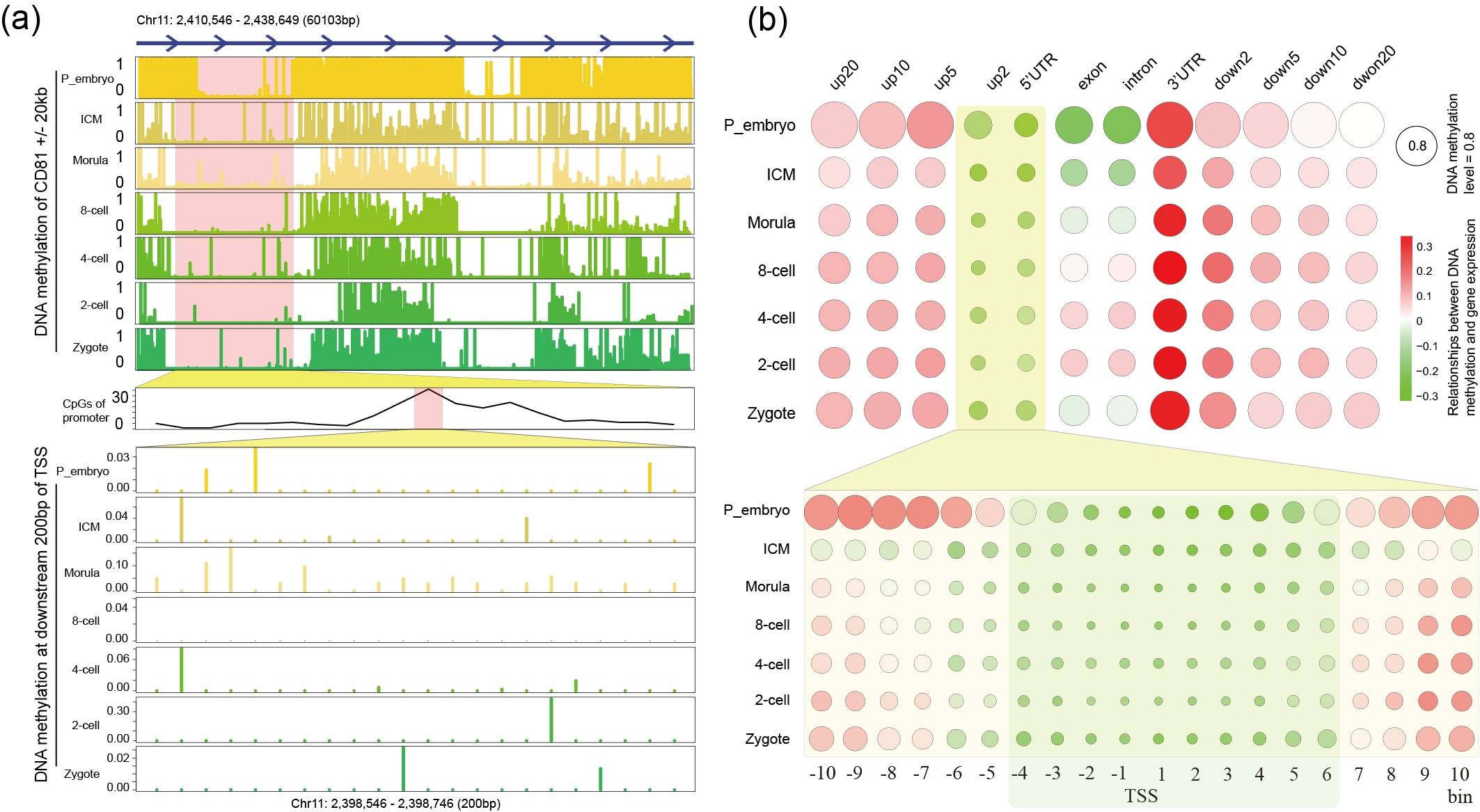

Supplement: figure S5 [file rsob180131supp5.tif]
