# Supplementary material for: DNA methylation subpatterns at distinct regulatory regions in human early embryos
Source: Open Biol. 2018 Oct 31;8(10):180131. doi: 10.1098/rsob.180131 (PMC6223221; doi:10.1098/rsob.180131)
Supplement: table S3.doc [file rsob180131supp8.doc]

**Supplementary Table S1.** Gene ontology Analysis of differentially methylated genes in bin 1.

| ID | Description(BP) | | N | *P* value |
| --- | --- | --- | --- | --- |
| GO:0006977 | DNA damage response, signal transduction by p53 class mediator resulting in cell cycle arrest | | 6 | 0.005846 |
| GO:0060045 | positive regulation of cardiac muscle cell proliferation | 4 | | 0.00753 |
| GO:0001525 | angiogenesis | 11 | | 0.009029 |
| GO:0046686 | response to cadmium ion | 4 | | 0.010795 |
| GO:0006120 | mitochondrial electron transport, NADH to ubiquinone | 5 | | 0.012852 |
| GO:0015992 | proton transport | 5 | | 0.012852 |
| GO:0008643 | carbohydrate transport | 4 | | 0.01336 |
| GO:0045931 | positive regulation of mitotic cell cycle | 4 | | 0.014762 |
| GO:0001666 | response to hypoxia | 9 | | 0.015227 |
| GO:0016477 | cell migration | 9 | | 0.015227 |
| GO:0045727 | positive regulation of translation | 5 | | 0.01677 |
| GO:0006364 | rRNA processing | 10 | | 0.018863 |
| GO:0001974 | blood vessel remodeling | 4 | | 0.02117 |
| GO:0032981 | mitochondrial respiratory chain complex I assembly | 5 | | 0.029573 |
| GO:0010667 | negative regulation of cardiac muscle cell apoptotic process | 3 | | 0.030807 |
| GO:1904707 | positive regulation of vascular smooth muscle cell proliferation | 3 | | 0.030807 |
| GO:0014068 | positive regulation of phosphatidylinositol 3-kinase signaling | 5 | | 0.032671 |
| GO:0051436 | negative regulation of ubiquitin-protein ligase activity involved in mitotic cell cycle | 5 | | 0.043062 |
| GO:0051298 | centrosome duplication | 3 | | 0.047799 |

Note. Only terms with more than two genes are shown. N is the number of genes. *P* values are corrected by Benjamini–Hochberg FDR.
